# Supplementary material for: Assessing Lifestyle in a Large Cohort of Undergraduate Students: Significance of Stress, Exercise and Nutrition
Source: Nutrients. 2024 Dec 16;16(24):4339. doi: 10.3390/nu16244339 (PMC11677779; doi:10.3390/nu16244339)

Figure S1: Association between the variables used to perform clustering according to MCA.

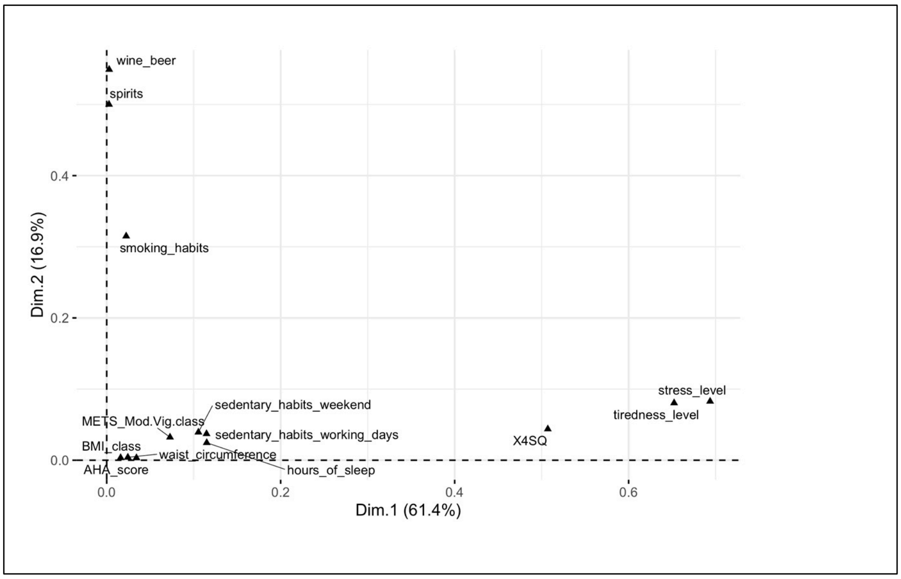

WC= Waist Circumference, BMI=Body Mass Index, METsMV= moderate and vigorous physical activity volume, habits_WD= Sedentary behavior during Working Days, habits_WE=Sedentary behavior during weekends, sleep_hours= hours of sleep, wine_beer= wine and beer consumption, spirits= spirit consumption, s4SQ= short questionnaire subjective somatic stress-related symptoms, AHA_Score=AHA Nutrition Score

Figure S2: average silhouette width to choose the number of clusters for the Kmodes algorithm.

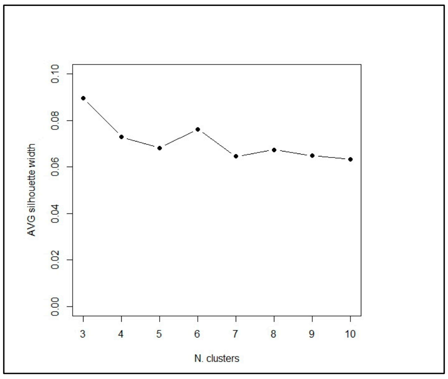

Supplement: Supplementary file 1 [file nutrients-16-04339-s001.zip › nutrients-3326986-supplementary.pdf]
